# Effects of Varying Dietary Lipid and Starch Levels on Growth Performance, Biochemical Components, and Hepatic Glycolipid Metabolism in Hybrid Grouper (*Epinephelus lanceolatus ♂* × *E. fuscoguttatus* ♀)

**DOI:** 10.3390/ani16091304

**Published:** 2026-04-23

**Authors:** Songhang Li, Kun Wang, Mengyao Chen, Yuan Li, Chong Wang, Kai Song, Yichuang Xu, Jidan Ye

**Affiliations:** Xiamen Key Laboratory for Feed Quality Testing and Safety Evaluation, Fisheries College, Jimei University, Xiamen 361021, China; 202312951055@jmu.edu.cn (S.L.); wangkun@jmu.edu.cn (K.W.); chenmy@jmu.edu.cn (M.C.); 202311908013@jmu.edu.cn (Y.L.); 202312951036@jmu.edu.cn (C.W.); songkai@jmu.edu.cn (K.S.); xuyichuang@jmu.edu.cn (Y.X.)

**Keywords:** blood biochemistry, body composition, energy metabolism, feed utilization, nutrition, aquaculture

## Abstract

A 56-day feeding trial was conducted to evaluate the effects of varying dietary lipid and starch levels on growth performance, biochemical components, and hepatic glycolipid metabolism in hybrid grouper. Nine isonitrogenous diets were formulated to contain three levels of lipid (6%, 10%, or 14%) and starch (14%, 21%, or 28%) using a 3 × 3 factorial design. Juvenile fish (initial body weight: 19.06 ± 0.03 g) were randomly allocated to 27 floating net cages (25 fish per cage, three replicates per diet) in an indoor seawater recirculation system and hand-fed to apparent satiation twice daily. Two-way ANOVA was conducted to check treatment effects of dietary lipid and starch levels. No interaction effect between lipid and starch on growth and feed utilization was observed across all treatments; however, significant interactions between the two were observed for condition factor (CF), and some serum biochemical indicators and some hepatic glycolipid metabolic enzyme activities. Growth rate, specific growth rate, and feed efficiency (FE) exhibited a declining trend with increasing dietary lipid levels (*p* < 0.05). Conversely, hepatosomatic index (HSI), viscerosomatic index (VSI), condition factor, hepatic lipid and glycogen contents, muscle lipid content, serum triglyceride and high-density lipoprotein cholesterol contents, as well as hepatic carnitine palmitoyltransferase 1 (CPT-1) and lipoprotein lipase (LPL) activities, showed an increasing trend (*p* < 0.05). As lipid levels increased, serum total cholesterol (TC) and total protein (TP) contents dropped to a minimum at the intermediate lipid level (10%) and then rose, regardless of starch level. Hepatic fructose-1,6-bisphosphatase (FBP) activity increased significantly when lipid level rose from 6% to 10% (*p* < 0.05). With increasing dietary starch levels, HSI, VSI, hepatic and muscle glycogen contents, and serum low-density lipoprotein cholesterol content increased, while FE and serum TP content decreased (*p* < 0.05). Hepatic CPT-1, LPL, FBP, and pyruvate kinase activities were significantly enhanced when starch levels increased from 14% to 21% or 28% (*p* < 0.05). Serum aspartate aminotransferase activity was significantly higher in fish fed 14% lipid compared to those fed 6% or 10% lipid. These findings indicate that there is no interaction of dietary lipid and starch on growth and feed utilization, but high dietary lipid (14%) may enhance hepatic lipid oxidation while suppressing glycolysis, thereby limiting growth and promoting hepatic lipid deposition. The results provide a practical reference for optimizing dietary lipid and starch levels in cost-effective feed formulations for hybrid grouper.

## Simple Summary

As non-protein energy sources, both lipid and starch play crucial roles in aquafeeds. Properly balancing these components not only maximizes dietary protein utilization for growth but also spares lipid for additional energy consumption, thereby preserving lipid for other essential physiological functions. Despite this nutritional and economic importance, research on the interactive effects of dietary lipid and starch on growth, feed utilization, and biochemical responses in fish remains limited. This knowledge gap is particularly pronounced for marine fish, such as hybrid grouper. This study therefore investigated how the fish adjusts its growth and intermediary metabolism in response to varying dietary levels of these two energy sources, thereby providing a scientific rationale for formulating more cost-effective feeds.

## 1. Introduction

Terrestrial farmed animals primarily utilize carbohydrates (mainly starch) as an energy source. In contrast, fish prioritize protein and lipid for energy supply [1,2]. This metabolic preference stems from evolutionary adaptation to carbohydrate-limited aquatic environments, which has shaped a distinct energy metabolism strategy [3]. However, under intensive aquaculture conditions, high-quality protein and lipid sources are increasingly limited and costly. Starch, by contrast, is widely available and produced in large quantities, creating a strong incentive to incorporate it into formulated fish feeds. Such inclusion can reduce the reliance on protein and lipid for energy, thereby exerting a protein- and lipid-sparing effect [4]. For instance, in European sea bass (*Dicentrarchus labrax*) [5], increasing gelatinized starch from 15% to 35% while reducing protein from 60% to 45% did not compromise growth performance, demonstrating significant protein-sparing capacity. Similarly, in gilthead sea bream (*Sparus aurata*) juveniles [6], decreasing dietary protein from 50% to 40% while increasing starch from 10% to 20% also had no adverse effect on growth performance. Moreover, fish fed the low-protein, high-starch diet exhibited improved protein efficiency ratio and nitrogen retention, alongside reduced nitrogen intake and economic conversion ratio, indicating improved protein utilization efficiency [4].

Although fish generally exhibit lower carbohydrate tolerance than terrestrial animals, they can utilize carbohydrates for energy metabolism when provided at appropriate levels [7]. When incorporated judiciously, starch can serve as an effective energy source, promoting growth and improving feed efficiency [8,9,10,11,12]. Nevertheless, the ability to utilize starch varies among species, with carnivorous fish showing particularly low utilization efficiency [7,13]. For example, hybrid grouper (*Epinephelus lanceolatus ♂* × *E. fuscoguttatus* ♀) can tolerate dietary starch levels up to 28% without compromising growth, but levels exceeding 20% lead to hepatic glycogen accumulation and metabolic stress [14]. Similarly, in largemouth bass (*Micropterus salmoides*), dietary starch exceeding 10% resulted in impaired growth and hepatic steatosis [15]. These results indicate that excessive dietary starch leads to growth retardation and metabolic disorders, including persistent hyperglycemia, hepatic glycogen accumulation, and fatty liver [16,17]. Elucidating the interactions between starch and protein or lipid, as well as their respective sparing effects, is essential for developing well-balanced feeds that enhance starch utilization and reduce production costs [11,12,18,19,20,21,22,23,24].

The hybrid grouper, a typical carnivorous marine species, is a major aquaculture fish in China due to its rapid growth, strong disease resistance, and high flesh quality [25]. Importantly, this hybrid exhibits a distinct metabolic phenotype characterized by impaired glucose tolerance and compromised carbohydrate utilization. Glucose administration induces prolonged hyperglycaemia (>12 h) in this species, making it an excellent model for studying starch-lipid interactions in carnivorous fish [26]. Furthermore, its relatively low tolerance to high-carbohydrate diets (>20%) leads to hepatic glycogen accumulation, fatty liver, and impaired non-specific immune function [27]. Consequently, these metabolic characteristics make the hybrid grouper particularly suitable for exploring how dietary lipids modulate starch utilization and glycolipid metabolism.

Given the hybrid grouper’s limited carbohydrate tolerance, dietary higher levels starch (21–28%) are likely to induce metabolic stress, manifested as glycogen accumulation and lipid deposition [26,28]. However, appropriate lipid supplementation can alleviate the metabolic burden caused by high-starch diets through activating fatty acid oxidation pathways and optimizing the starch-to-lipid ratio [8]. Although previous studies have established optimal dietary protein (36–51%) and lipid levels (10–16%) [29,30], as well as starch-to-lipid ratios (0.77–2.27) [8], and a protein-sparing effect of starch has been observed in orange-spotted grouper (*Epinephelus coioides*) fed 45% protein with 20% corn starch [31], these investigations largely employed single-factor designs in which starch and lipid levels were inversely adjusted [8]. Moreover, many studies have determined appropriate levels of lipid and starch in feed based on their interactive effects on growth performance so far [22,32,33]. However, little information is still available regarding the interactive effects of dietary lipid and starch on glycolipid metabolism in farmed fish, including hybrid grouper. Therefore, this study aimed to investigate how different dietary lipid and starch levels affect the growth performance and blood biochemical indicators by interactively regulate hepatic glycolipid metabolism in hybrid grouper, using ranges of lipid (6–14%) and starch (14–28%) that are suitable for hybrid grouper growth as determined from cultivation practices.

## 2. Materials and Methods

### 2.1. Diet Preparation

Corn is the most important feed energy ingredient globally [34], and corn starch has been widely used as an energy source and filler in fish feeds in China [3]. Nine isonitrogenous experimental diets were formulated with three lipid levels (6%, 10%, and 14%) and three starch levels (14%, 21%, and 28%), using corn starch as the sole starch source, a mixture of soy oil and fish oil along with soy lecithin as the primary lipid sources, and fish meal, soy protein isolate, casein, gelatin, and wheat gluten as the main protein sources (Table 1). The nine diets were designated as D1 (L6/S14), D2 (L6/S21), D3 (L6/S28), D4 (L10/S14), D5 (L10/S21), D6 (L10/S28), D7 (L14/S14), D8 (L14/S21), and D9 (L14/S28), corresponding to their lipid and starch concentrations. The preparation of experimental diets followed Chen’s method [35]. Briefly, solid raw ingredients were ground and passed through a 60-mesh sieve. All powdered and lipid ingredients were weighed, manually blended, and mixed with an appropriate amount of water to form a soft dough by kneading. The dough was extruded through 2.5 mm and 4 mm dies to produce moist pellets, which were then dried in a ventilated oven at 55 °C for 24 h, following the method described by Qin’s method [36]. The resulting dry feed was sealed in plastic bags and stored at −20 °C until use.

### 2.2. Daily Feeding and Management

Juvenile hybrid groupers were obtained from Yichang Aquaculture Co., Ltd. (Dongshan County, Zhangzhou, China) and temporarily held in a cement pool (30 m^2^) for a two-week acclimation period, during which they were fed a commercial diet (49.1% crude protein, 11.7% crude lipid, and 9.5% moisture). After 24 h of fasting, fish of similar size (initial body weight: 19.06 ± 0.03 g/fish) were captured and randomly allocated into 27 floating net cages (1.2 m length × 0.8 m width × 0.5 m height) with mesh covers, each stocked with 25 fish. The cages were placed inside an indoor pool (10 m length × 8 m width × 1.8 m depth), evenly spaced along the pool edge and secured with ropes at each corner. Continuous aeration was provided via perforated pipes fixed at 1 m intervals along the pool bottom. The growth trial was conducted over eight weeks, with fish randomly assigned to nine dietary groups, each with three replicate cages. Fish were hand-fed to apparent satiation twice daily (07:30 and 17:30) under a natural photoperiod. A tray (0.8 m × 0.6 m) placed at the bottom of each cage collected uneaten feed, which was retrieved 30 min after feeding, dried at 65 °C, and weighed to determine actual feed intake. Leaching loss was corrected by subtracting the weight of feed recovered from a blank cage exposed to identical conditions. Actual feed intake was calculated as the difference between feed offered and corrected uneaten feed. The pool was cleaned weekly, and approximately three-fifths of the seawater was renewed after each cleaning. Throughout the feeding trial, the rearing pool conditions were maintained as follows: seawater temperature, 25.7–30.3 °C; dissolved oxygen, 6.1–8.2 mg/L; pH, 7.9–8.3; ammonia-nitrogen, 0.1–0.3 mg/L; and salinity, 29–33. This experimental protocol was approved by Jimei University Laboratory Animal Ethics Committee (the approval number: 2011-58).

### 2.3. Sampling Procedure and Index Measurement

At the conclusion of the growth trial, fish were fasted for 24 h according to the protocol described by Özel et al. [37]. All fish from each cage were then captured using a net, bulk-weighed, and counted to calculate growth rate (GR), specific growth rate (SGR), feed efficiency (FE), and survival rate (SR). Thirty-three fish per dietary group (eleven per replicate cage) were randomly captured and anesthetized with 100 mg/L MS-222 (tricaine methanesulfonate, Macklin Biochemical Technology Co., Ltd., Shanghai, China). Individual body weight and length were recorded, and blood samples were collected from the caudal vein using 2 mL syringes. Blood was allowed to clot at 4 °C for 12 h, then centrifuged at 1027× *g* for 10 min at 4 °C to obtain serum. The collected serum from the 11 fish in each cage was pooled, immediately frozen in liquid nitrogen, and then stored at −80 °C. The total serum volume was sufficient for the subsequent determination of biochemical indicators. Following blood collection, liver and viscera were aseptically removed to determine hepatosomatic index (HSI), viscerosomatic index (VSI), and condition factor (CF). Dorsal muscle samples were also collected. The liver and muscle samples were pooled by cage respectively and placed into 10 mL tubes, immediately frozen in liquid nitrogen, and then stored at −80 °C for determination of proximate composition and hepatic glycolipid metabolism enzyme activities.

### 2.4. Index Measurement

Liver and muscle samples were homogenized and freeze-dried. Glycogen content in these tissues was measured using commercial kits (Nanjing Jiancheng Bioengineering Co., Ltd., A043-1-1, Nanjing, China). Moisture, crude protein, and crude lipid in feed, and liver and muscle samples were analyzed following methods described by Chen et al. [35]. Gross energy was determined using an automatic calorimeter (PARR6300, Parr Instrument Company, Moline, IL, USA). Total starch was determined via the GOPOD method using kit (K-TSTA-50A, Megazyme Ltd., Bray, Wicklow, Ireland). Glycogen was determined via KOH/anthrone method using a multifunctional microplate reader (Infinite 200pro, Shanghai, China).

Supernatant of liver homogenates was collected via centrifugation for enzyme activity assay. Pyruvate kinase (PK, A076-1-2), phosphofructokinase (PFK, A129-1-1), phosphoenolpyruvate carboxykinase (PEPCK, A131-1-1), fructose-1,6-diphosphatase (FBP, A160-1-1), total protein (TP, A045-4), total cholesterol (TC, A111-1-1), high-density lipoprotein cholesterol (HDL-C, A112-1-1), low-density lipoprotein cholesterol (LDL-C, A113-1-1), triglycerides (TG, A110-1-1), alanine aminotransferase (ALT, C009-2-1), and aspartate aminotransferase (AST, C010-2-1) were measured using kits (Nanjing Jiancheng Bioengineering Co., Ltd.), while carnitine palmitoyltransferase 1 (CPT-1, MM-1030F1), fatty acid synthase (FAS, MM-32945O1), and lipoprotein lipase (LPL, MM-32946O1) were measured using kits (Jiangsu Enzyme-Immunoassay Biotechnology Co., Ltd., Yancheng, China). TG and TC were measured using the COD-PAP method, while TP was determined via the biuret method. ALT and AST were assessed using the Reitman-Frankel method, and HDL-C and LDL-C were measured by the peroxidase colorimetric method. All of these assays were performed using the microplate reader (Infinite 200 Pro). CPT-1, LPL, and FAS were quantified using a double-antibody sandwich ELISA, also conducted on the microplate reader (Infinite 200 Pro). PK, PEPCK, and PFK were measured using the NADH rate method, and FBP was determined by the NADPH generation method, with all measurements carried out on a visible spectrophotometer (V-1100D, MAPADA, Shanghai, China).

### 2.5. Calculations

The parameters of growth performance were calculated as the following equations:

Growth rate (GR, %) = 100% × (W2 − W1)/W1

Specific growth rate (SGR, %/day) = 100% × (ln W2 – ln W1)/t

Feed efficiency (FE) = (W2 − W1)/F

Survival rate (SR, %) = 100% × N2/N1

Hepatosomatic index (HSI, %) = 100% × Wh/W

Viscerosomatic index (VSI, %) = 100% × Wv/W

Condition Factor (CF, g/cm^3^) = 100% × W/L^3^

where W1, initial body weight (g/fish); W2, final body weight (g/fish); F, feed intake (g/fish); t, trial duration(days); N1, initial fish number; N2, final fish number; W, individual body weight (g); L, individual body length (cm); Wh, individual liver weight (g); Wv, individual visceral weight (g).

### 2.6. Statistical Analysis

All data are presented as mean ± standard error. Normality and homogeneity of variances were verified using Kolmogorov–Smirnov and Levene’s tests, respectively. Data were first analyzed using one-way ANOVA to detect differences among all nine treatments. A two-way ANOVA was used to test the effects of lipid and starch levels on growth performance and biochemical indicators, followed by Duncan’s multiple range test for post hoc comparisons. Data expressed as percentages were subjected to arcsine transformation before statistical analysis. Statistical analyses were performed using SPSS v27.0 (IBM Corp., Armonk, NY, USA). Differences were considered statistically significant at *p* < 0.05.

## 3. Results

### 3.1. Growth Performance

**Figure 1:**
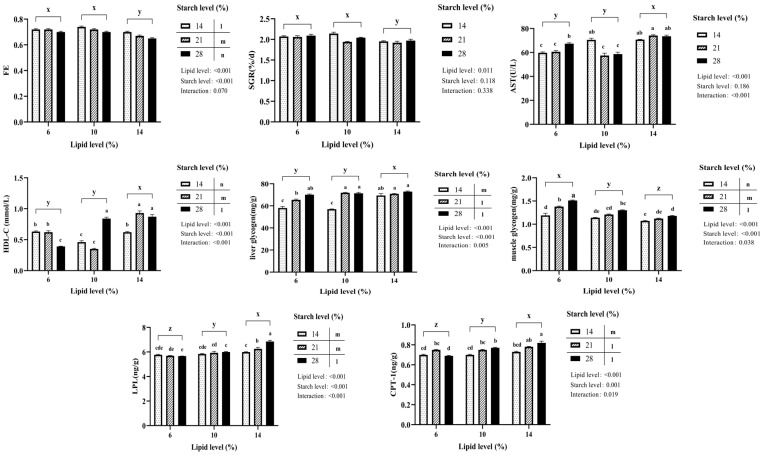
Effects of varying dietary lipid and starch levels on SGR, FE, serum AST and HDL-C levels, liver and muscle glycogen contents, and liver CPT-1 and LPL activities in hybrid grouper. Data are expressed as mean ± SE. Bars bearing the different lowercase letters indicate significant differences (*p* < 0.05). Bars with different superscripts (x, y and z) indicate significant differences (*p* < 0.05) within the lipid diet groups, regardless of dietary starch level. Bars with different superscripts (l, m and n) indicate significant differences (*p* < 0.05) within the starch diet groups, regardless of dietary lipid level. SGR, specific growth rate; FE, feed efficiency; HDL-C, high-density lipoprotein cholesterol; AST, aspartate aminotransferase; CPT-1, carnitine palmitoyltransferase 1; LPL, lipoprotein lipase.

As shown in Table 2, dietary lipid and starch levels did not significantly affect GR, SGR, FE, HSI, or SR among all treatments (*p* > 0.05; Figure 1), but significantly influenced VSI and CF (*p* < 0.05). Regardless of starch level, increasing lipid levels led to a decline in GR, SGR, and FE, while HSI, VSI, and CF showed the opposite trend (*p* < 0.05). Increasing starch levels, irrespective of lipid level, resulted in decreased FE but increased HSI and VSI (*p* < 0.05). No interactions between lipid and starch levels were detected for growth performance indicators, except for CF. Similar CF values were found for diet L6/S28 versus diet L10/S14, and for diet L10/S21 versus diet L14/S14 (*p* > 0.05).

### 3.2. Liver and Muscle Composition

Dietary lipid and starch levels significantly influenced hepatic protein and glycogen content among all treatments (*p* < 0.05; Table 3 and Figure 1). Regardless of starch level, liver lipid and glycogen content increased with higher dietary lipid levels, whereas liver protein content decreased. Glycogen content increased with higher starch levels, an effect independent of dietary lipid concentration. A significant interaction was observed between dietary lipid and starch levels on liver protein and glycogen content (*p* < 0.05). For instance, at the lipid level of 6%, liver protein contents were higher than those at the 10% or 14% lipid levels in response to starch levels (*p* < 0.05). Similar glycogen contents were observed between diet L6/S28 and diet L10/S21, as well as between diet L10/S28 and diet L14/S14 (*p* > 0.05).

Muscle lipid, protein, and glycogen content were also significantly affected by dietary treatments across all treatments (*p* < 0.05; Figure 1). Muscle lipid content increased with higher lipid level (14%), whereas glycogen content decreased, a pattern independent of starch level. Regarding starch, glycogen content rose with increasing starch. Conversely, lipid content initially remained stable but then showed a significant decline at the 28% starch level (*p* < 0.05). Significant interactive effects of dietary lipid and starch were observed on muscle lipid, protein, and glycogen contents (*p* < 0.05). At the 6% lipid level, muscle lipid contents were lower than those at the 10% and 14% lipid in response to starch levels (*p* < 0.05). Similar protein values were noted between diet L6/S21 and diet L10/S14, as well as between diet L10/S28 and diet L10/S14 (*p* > 0.05). At the lipid level of 6%, muscle glycogen contents were higher than those at the 10% or 14% lipid levels in response to starch levels (*p* < 0.05)

### 3.3. Serum Parameters

Dietary lipid and starch levels significantly influenced serum TG, TP, AST and HDL-C concentrations (*p* < 0.05), while no significant effects were observed on TC, ALT and LDL-C across all treatments (*p* > 0.05; Table 4 and Figure 1). Regardless of dietary starch level, serum TG exhibited an overall increasing trend with elevated lipid levels. In contrast, TP dropped to a minimum at the intermediate lipid level (10%) and then rose, while AST and HDL-C remained initially stable before increasing significantly at the 14% lipid level (*p* < 0.05). Regarding lipid level, TC dropped to a minimum at 21% starch, TP decreased as starch level increased, while HDL-C demonstrated a significant concurrent elevation (*p* < 0.05). Significant interactions were observed for TG, TP, AST and HDL-C between lipid and starch (*p* < 0.05). The TG and TP values were similarly noted between diet L6/S28 and diet L10/S21, as well as between diet L10/S28 and diet L14/S14 (*p* > 0.05). Likewise, comparable AST values were observed between diet L6/S28 and diet L10/S14 (*p* > 0.05). Regarding HDL-C value, diet L6/S28 was similar to diet L10/S14, and diet L10/S28 was comparable to diet L10/S21 (*p* > 0.05).

### 3.4. Hepatic Glycolipid Metabolism

The activities of CPT-1, LPL, FBP, and PK in the liver were significantly influenced by dietary lipid and starch levels (*p* < 0.05; Table 5 and Figure 1), whereas FAS, PEPCK, and PFK activities showed no significant alterations across all treatments (*p* > 0.05; Table 5). Irrespective of dietary starch level, CPT-1 and LPL activities increased significantly with elevated lipid levels (*p* < 0.05), whereas FAS, PK, and PFK activities decreased. FBP activity showed an initial increase followed by stabilization, with activity at the 6% lipid level being significantly lower than at the 10% and 14% levels (*p* < 0.05). Regarding starch level, increased dietary starch content enhanced the activities of CPT-1, LPL, FBP, and PK but suppressed PEPCK. Significant interactions were observed for CPT-1, LPL, FBP, and PK between lipid and starch (*p* < 0.05). For instance, comparable CPT-1 activity was noted between diet L10/S28 and diet L10/S14 or diet L14/S14 (*p* > 0.05). Similar LPL values were noted between diet L6/S28 and diet L10/S14, as well as between diet L10/S28 and diet L14/S14 (*p* > 0.05). Similar FBP values were observed for diet L6/S28 versus diet L14/S14 (*p* > 0.05). Similar PK values were noted between diet L6/S28 and diet L10/S21, and between diet L10/S28 and diet L14/S14 (*p* > 0.05).

## 4. Discussion

An unexpected result of this study is that varying dietary lipid (6–14%) and starch (14–28%) levels did not significantly affect the growth performance or feed utilization of hybrid grouper, which is consistent with findings in mandarin fish (*Siniperca chuatsi*) [22,32] and large yellow croaker (*Larmichthys crocea*) [22,33]. This may be attributed to the fact that the nutritional levels of lipid and starch used in these experiments already met the growth requirements of these fish. When protein and non-protein energy (such as lipid and starch) were sufficient, carnivorous fish could maintain normal growth performance through metabolic regulation [3]. This was accompanied by an improved metabolic balance between lipid and starch [32], reflecting a synergistic growth-promoting effect of lipid and starch [5,8,22]. Moreover, the high lipid level (14%) in this study may have attenuated the potential effects of starch on hepatic glycogen deposition. This aligns with Han et al. [32], who found that high lipid levels (12–16%) promoted hepatic glycogen deposition rather than enhancing lipid oxidation in mandarin fish, as fish prioritized lipid oxidation for energy supply, while starch was stored in the form of glycogen [22,32].

Although no interactive effects were observed, the individual effects of lipid and starch were significant. The GR, SGR, and FE declined with increasing lipid levels, and FE also decreased with higher starch levels. These results were consistent with the findings of Xu et al. [38], and further supported by evidence that a high-lipid diet significantly lowers protein and energy retention efficiency as well as feed efficiency in European sea bass [39] and that a high-starch diet reduces intestinal amylase and trypsin activities in cobia (*Rachycentron canadumas*) [40]. Notably, HSI, VSI, and CF increased with elevated dietary lipid or starch alone, indicating altered body condition without compromising growth. Similar trends have been reported in other fish species [41,42,43,44,45,46,47,48], suggesting that excessive dietary lipid or starch may induce metabolic changes affecting physical fitness [8,49].

In this study, a significant interaction between lipid and starch levels was observed on CF. However, this significant change is mainly due to the influence of dietary lipid levels rather than starch levels, as there is no lipid–starch interaction effect on liver lipid (Table 3). Hepatic lipid deposition increased markedly with dietary lipid levels, while protein deposition decreased and glycogen accumulated. This aligns with previous studies [50,51,52] and indicates that lipid level exerts a stronger influence than starch on hepatic lipid storage in hybrid grouper [8]. Carnivorous fish have good adaptability to high-protein and high-lipid diets [3]; they can prioritize lipid oxidation for energy supply [53], and deposit excessive energy in the form of lipid in the body, including the liver [54]. Under high starch intake, excess glucose is mainly converted to glycogen or/and lipid by fish [22], due to limited glucose oxidation capacity [55,56]. This may account for the synergistic promotion of body lipid deposit by starch and lipid at high lipid levels, thereby enhancing VSI and/or CF [32,57,58]. Elevated lipid deposition likely results from upregulated lipogenesis or reduced fatty acid oxidation [59,60], as a result of excessive lipid supply.

Hepatic glycogen increased with both lipid and starch levels, consistent with earlier reports [14,17,61]. This result was attributed to the synergistic promotion by starch and lipid [8,62]; excessive fatty acids and glucose promote glycogen deposit through gluconeogenesis and glycogen synthesis [8,63]. Notably, fish fed diets with 6% lipid and 28% starch, 10% lipid and 21–28% starch, or 14% lipid and 14–28% starch showed comparable hepatic glycogen levels, indicating a synergistic interaction [22,64]. These results suggest that dietary lipid and starch jointly promote hepatic glycogen accumulation.

Muscle lipid content increased with dietary lipid levels, likely due to enhanced fatty acid influx or endogenous lipogenesis [49,57,65]. However, the high starch level (28%) lowered muscle lipid content compared to the other two starch levels (14% and 21%). This may be explained by the energy allocation strategy adopted by the fish in response to high-starch diets: high starch diets enhances muscle glycolytic capacity while simultaneously prioritizing glycogen synthesis in the liver and visceral adipose tissue, thereby increasing energy production via muscle lipid oxidation and reducing lipid deposition [44,66,67]. In contrast, muscle glycogen decreased with increasing lipid but increased with starch, suggesting that lipid-rich diets favor lipid oxidation, whereas starch-rich diets promote glycogen storage [22,50,68]. The reduction in muscle glycogen under high lipid conditions may reflect metabolic prioritization of lipid over carbohydrate as an energy source [65,69,70].

Muscle protein remained relatively stable across treatments, likely due to the fixed dietary protein level and the structural role of muscle tissue, which is less responsive to short-term dietary modulation [11,22,71]. Furthermore, while the main effects were not significant, the interaction effect on muscle protein deposition was significant. This finding aligns with a study on mandarin fish [32]. When lipid levels are low, starch exerts a protein-sparing effect by providing auxiliary energy. However, at higher lipid levels, the synergistic energy provision from starch and lipid leads to energy surplus, subsequently favoring lipid deposition over muscle protein synthesis [32,50].

Serum parameters are key indicators of nutritional and health status [3,12], and lower or higher serum contents will mirror abnormal physiological and biochemical changes that occur in animals. Serum TG increased with dietary lipid level, consistent with findings in large yellow croaker [72]. Although starch alone did not affect TG, a significant lipid–starch interaction was observed, suggesting that carbohydrate-derived energy may promote lipogenesis under moderate lipid supply [5]. Dietary starch levels had a significant effect on TC, with the lowest TC recorded at 21% starch. This is aligns with Chen et al. [73], who reported that moderate starch levels optimize glycolipid metabolism and reduce serum TC. The underlying mechanism may involve the upregulation of insulin signaling and glycolytic enzyme gene expression, alongside the downregulation of gluconeogenic enzyme genes, thereby improving the metabolic balance between starch and lipid [32]. At 28% starch, TC returned to a level comparable to that at 14% starch, similar to findings by Liu et al. [74], where plasma TC increased significantly when starch levels exceeded 21% (i.e., at 28% and 35%), possibly reflecting cholesterol synthesis induced by excessive carbohydrate intake [75].

Higher lipid levels elevated HDL-C, and increasing starch further enhanced this effect, particularly at the 14% lipid, indicating improved cholesterol transport. Diets high in either lipid or starch alone have also been shown to promote serum HDL-C in largemouth bass [76] and kelp grouper (*Epinephelus moara*) [57]. Moreover, an interaction effect of dietary starch and lipid on serum HDL-C has been reported in gilthead seabream [77].

Serum TP was significantly influenced by the interactive effect of lipid and starch levels. TP initially decreased and then increased with rising lipid levels, whereas, it decreased as starch levels increased. This clear interaction reflects the metabolic characteristic of carnivorous fish, which preferentially utilize lipid for energy [78]. Compared with the other two combinations, the low-lipid (6%) and high-starch (28%) combination resulted in lower serum TP, indicating insufficient energy supply from lipid and a compensatory increase in energy from starch. Consequently, reduced energy metabolism density limits hepatic protein synthesis, leading to decrease TP [79]; At the moderate lipid level (10%), an imbalance between glycolysis and fatty acid oxidation for energy provision may impose a significant metabolic load on the liver, further reducing TP production reduction due to insufficient energy supply [41]; at the high lipid level (14%), lipid became the primary energy source, mitigating the negative effects of starch on TP content, and TP remained stable regardless of starch levels [80].

Serum AST activity was significantly influenced by the interaction between lipid and starch levels. At 6% lipid, AST activity increased with rising starch level, suggesting that high starch induces metabolic stress under low-lipid conditions [17]. Elevated AST at 14% lipid suggests increased hepatic metabolic demand or mild hepatocyte stress induced by excess fatty acids [8,81]. Notably, the combination of 10% lipid and 21% starch resulted in the lowest AST activity, representing an optimal metabolic balance point. This finding aligns with the nutritional interaction effects of starch and lipid observed in gilthead seabream [82], where nutrient combinations, rather than single factors, trigger adaptive changes in hepatic metabolic enzymes [68].

In this study, the hepatic activities of key enzymes involved in lipid metabolism—namely CPT-1, FAS, and LPL—were measured to assess the effects of dietary lipid and starch levels in hybrid grouper. CPT-1 facilitates the transport of fatty acids into mitochondria for β-oxidation. Under conditions of sufficient cellular energy, CPT-1 activity is inhibited, suppressing fatty acid oxidation and shifting metabolic flux toward fatty acid synthesis [83]. FAS is a key enzyme responsible for the de novo synthesis of long-chain fatty acids [84], while LPL hydrolyzes TG present in very-low-density lipoproteins and chylomicrons, thereby regulating lipid uptake and distribution [85]. Hepatic CPT-1 and LPL activities increased with lipid level, indicating enhanced fatty acid oxidation and lipid turnover, while FAS remained unchanged, suggesting that high-lipid diets promote lipid utilization over de novo synthesis [86]. Elevated CPT-1 and LPL at higher starch levels, without corresponding lipid accumulation, implies that starch-induced metabolic regulation favors oxidation over lipogenesis [87].

Regarding glucose metabolism, FBP and PEPCK are rate-limiting enzymes for gluconeogenesis and are primarily activated when endogenous glucose production is required [88]. FBP activity increased with lipid and starch, indicating enhanced gluconeogenesis, while PEPCK decreased with starch, reflecting reduced reliance on gluconeogenic precursors when dietary carbohydrate is sufficient [89,90,91]. PK and PFK activities were suppressed by high lipid but enhanced by high starch, suggesting that lipid-derived energy reduces glycolytic demand, while starch loading increases glycolytic flux [92,93]. Excess glucose is consequently stored as hepatic glycogen [51].

Significant interactions between lipid and starch levels were observed for the glycolytic enzymes CPT-1, LPL, FBP, and PK. The activities of these enzymes were similar under two dietary contrasting conditions: low lipid combined with high starch (L6/S28) and medium lipid combined with low starch (L10/S14), as well as between medium lipid combined with high starch (L10/S28) and high lipid combined with low starch (L14/S14). These patterns reflect a lipid-sparing effect caused by starch. On the one hand, at the low lipid level of 6%, starch exerted a more pronounced influence on these enzymes, highlighting the dominant role of carbohydrates in metabolic regulation under energy-limited conditions. However, the growth achieved with the low-lipid and high-starch diet essentially came at the cost of high metabolic stress, as indicated by elevated AST levels. As noted earlier, at a high lipid level of 14%, lipid mitigated the negative effects of starch on hepatic glycogen deposition, accompanied by increased CF, VSI, and hepatic lipid content, along with suppressed glycolytic pathway (lowered PK). This aligns with findings in barramundi (*Lates calcarifer*) [94], where decreasing starch-to-lipid ratios increased fatty acid oxidation enzyme activity and inhibited glycolysis enzyme activity, suggesting that muscle triacylglycerol-bound fatty acid synthesis represents the primary pathway for starch-induced lipid deposition. On the other hand, high-lipid combined with high-starch diets (extreme combinations) could promote metabolic dysregulation, potentially leading to insulin resistance and fatty liver disease with chronic exposure [95,96]. Notably, the 10L/14S diet exhibited higher PK activity than 10L/21S diet, indicating that 14% starch was insufficient to maintain an effective glycolytic flux. In contrast to 10L/21S diet, 10L/28S diet marginally reduced PK activity. Overall, a medium-lipid and medium-starch (L10/S21) diet appears to favor the maintenance of glycolipid metabolic balance, exhibiting moderate activities of CPT-1, LPL, and PK that satisfy energy demands without inducing metabolic stress.

## 5. Conclusions

Although no interactive effects of dietary lipid and starch levels on growth rate and feed utilization in hybrid grouper, lipid and starch alone markedly influenced growth performance, tissue composition, serum parameters, and hepatic glycolipid metabolic enzyme activities. A combination of 10% lipid and 21% starch achieved an optimal balance of glycolipid metabolism while minimizing metabolic stress. Specifically, high lipid (14%) enhanced fatty acid oxidation and suppressed glycolysis, increasing metabolic load, while high starch (28%) promoted hepatic glycogen deposition but reduced feed efficiency. Synergistic effects between lipid and starch were observed in energy metabolism, highlighting the need for balanced formulation to optimize health and growth performance in this species.

The present study focuses on the interaction effects of lipid and starch on performance and physiological parameters on the fish at the grow-out stage. Giving the relatively short experimental duration and controlled laboratory conditions, these findings may not fully reflect long-term outcomes or the complexities of commercial aquaculture. More importantly, determining their appropriate inclusion levels of lipid and starch during the finishing stage is crucial for the practical application of feed formulations across in the entire production cycle. This aspect will be a key focus of future research.

## Figures and Tables

**Table 1:** Ingredients and nutrient levels of the experimental diets.

Ingredients (%)	D1 (L6/S14)	D2 (L6/S21)	D3 (L6/S28)	D4 (L10/S14)	D5 (L10/S21)	D6 (L10/S28)	D7 (L14/S14)	D8 (L14/S21)	D9 (L14/S28)
Fish meal	20	20	20	20	20	20	20	20	20
Soy protein isolate	19	19	19	19	19	19	19	19	19
Wheat gluten	7.5	7.5	7.5	7.5	7.5	7.5	7.5	7.5	7.5
Casein	9.8	9.8	9.8	9.8	9.8	9.8	9.8	9.8	9.8
Gelatin	2	2	2	2	2	2	2	2	2
Peru fish/soy oil (1:1)	1.33	1.33	1.33	5.43	5.43	5.43	9.53	9.53	9.53
Soy lecithin	2	2	2	2	2	2	2	2	2
Corn starch	14	21	28	14	21	28	14	21	28
Vitamin premix	0.3	0.3	0.3	0.3	0.3	0.3	0.3	0.3	0.3
Mineral premix	0.2	0.2	0.2	0.2	0.2	0.2	0.2	0.2	0.2
Choline chloride	0.1	0.1	0.1	0.1	0.1	0.1	0.1	0.1	0.1
Mold inhibitor	0.05	0.05	0.05	0.05	0.05	0.05	0.05	0.05	0.05
Ca(H_2_PO_4_)_2_	1.5	1.5	1.5	1.5	1.5	1.5	1.5	1.5	1.5
Stay-C 35%	0.02	0.02	0.02	0.02	0.02	0.02	0.02	0.02	0.02
Zeolite	11.1	7.6	4.1	9.05	5.55	2.05	7	3.5	0
Cellulose	11.1	7.6	4.1	9.05	5.55	2.05	7	3.5	0
Nutrient levels									
DM (%)	96.09	95.78	95.41	95.58	95.33	95.09	95.38	95.04	94.72
CP (%)	46.2	45.9	45.9	46.1	46.1	46.2	46.2	46.2	45.9
CL (%)	6.19	6.16	6.08	10.06	10.02	10.18	14.09	14.15	13.97
Ash (%)	14.72	11.82	8.83	13.11	10.03	7.09	11.25	8.29	5.31
NFE (%)	15.88	23.51	30.32	15.69	23.14	30.07	16.26	23.45	30.93
Starch (%)	14.25	21.72	28.46	14.13	21.44	28.21	14.36	21.64	28.91
GE (MJ/kg)	17.98	18.74	19.52	19.31	20.13	20.98	20.72	21.51	22.27
DE (MJ/kg)	14.03	14.67	15.38	15.43	16.02	16.77	16.80	17.47	18.03

DM, dry matter; CP, crude protein; CL, crude lipid; NFE, nitrogen-free extract; GE, gross energy; DE, digestible energy. Casein and gelatin were provided from Hualing Dairy Co., Ltd., Hezou, China. All other feed ingredients were sourced from Jiakang Feed Co., Ltd., Xiamen, China The composition of vitamin and trace mineral premixes was listed as specified in [35].

**Table 2:** Effects of varying dietary lipid and starch levels on growth performance in hybrid grouper.

Lipid Level (%)	Starch Level (%)	IBW (g/Fish)	FBW (g/Fish)	SR (%)	GR (%)	HSI (%)	VSI (%)	CF (g/cm^3^)
6	14	19.05 ± 0.06	64.33 ± 0.98	97.33 ± 1.33	194.23 ± 3.68	3.10 ± 0.08	7.39 ± 0.17 ^d^	2.74 ± 0.09 ^b^
	21	18.96 ± 0.06	63.60 ± 1.83	100.00 ± 0.00	204.17 ± 11.15	3.79 ± 0.23	8.25 ± 0.36 ^cd^	2.43 ± 0.08 ^c^
	28	18.98 ± 0.07	64.00 ± 1.66	97.33 ± 1.33	197.21 ± 9.20	3.73 ± 0.19	8.23 ± 0.35 ^cd^	2.37 ± 0.02 ^c^
10	14	19.15 ± 0.08	64.87 ± 2.78	96.00 ± 0.00	195.76 ± 14.44	3.49 ± 0.20	8.56 ± 0.35 ^bc^	2.34 ± 0.04 ^c^
	21	19.23 ± 0.09	58.60 ± 1.50	100.00 ± 0.00	173.40 ± 3.89	3.80 ± 0.25	8.92 ± 0.32 ^abc^	2.25 ± 0.02 ^c^
	28	19.11 ± 0.10	62.73 ± 0.73	97.33 ± 1.33	200.52 ± 3.11	4.09 ± 0.20	9.17 ± 0.34 ^abc^	2.28 ± 0.08 ^c^
14	14	19.08 ± 0.09	59.97 ± 1.15	100.00 ± 0.00	178.36 ± 8.66	3.68 ± 0.01	8.86 ± 0.20 ^abc^	2.43 ± 0.12 ^c^
	21	19.00 ± 0.05	58.90 ± 1.92	100.00 ± 0.00	173.71 ± 8.93	4.12 ± 0.31	9.61 ± 0.29 ^a^	2.89 ± 0.11 ^b^
	28	18.98 ± 0.08	59.17 ± 2.78	98.67 ± 0.67	185.87 ± 14.67	4.03 ± 0.16	9.51 ± 0.34 ^ab^	3.34 ± 0.13 ^a^
Main effects								
Lipid level	6				198.54 ± 4.55 ^x^	3.54 ± 0.14	7.95 ± 0.21 ^y^	2.51 ± 0.7 ^y^
	10				189.89 ± 5.82 ^xy^	3.79 ± 0.14	8.88 ± 0.18 ^x^	2.29 ± 0.03 ^z^
	14				179.32 ± 5.83 ^y^	3.95 ± 0.12	9.32 ± 0.18 ^x^	2.89 ± 0.14 ^x^
Starch level	14				189.45 ± 5.42	3.42 ± 0.11 ^m^	8.27 ± 0.24 ^m^	2.50 ± 0.08
	21				183.76 ± 6.66	3.90 ± 0.14 ^L^	8.92 ± 0.25 ^L^	2.52 ± 0.10
	28				194.53 ± 5.54	3.95 ± 0.11 ^L^	8.97 ± 0.26 ^L^	2.66 ± 0.18
Two-way ANOVA (*p*-value)								
Lipid level					0.036	0.066	<0.001	<0.001
Starch level					0.306	0.008	0.015	0.060
Interaction					0.289	0.774	0.915	<0.001

IBW, initial body weight; FBW, final body weight; GR, growth rate; HSI, hepatosomatic index; VSI, viscerosomatic index; CF, condition factor. Values with different superscript letters in the each column were significantly different (*p* < 0.05). Data are expressed as means ± SE.

**Table 3:** Effects of varying dietary lipid and starch levels on liver and muscle composition (%) in hybrid grouper.

Lipid Level (%)	Starch Level (%)	Liver			Muscle		
		Moisture	Lipid	Protein	Moisture	Lipid	Protein
6	14	62.95 ± 1.11	9.15 ± 0.10	17.70 ± 0.25 ^a^	74.94 ± 0.14	7.45 ± 0.24 ^e^	20.92 ± 0.21 ^c^
	21	63.81 ± 2.18	8.77 ± 0.31	16.10 ± 0.15 ^b^	74.63 ± 0.08	7.37 ± 0.12 ^e^	21.38 ± 0.02 ^b^
	28	64.75 ± 0.58	9.18 ± 0.25	15.23 ± 0.20 ^c^	74.77 ± 0.16	6.60 ± 0.09 ^f^	21.82 ± 0.01 ^a^
10	14	64.06 ± 0.58	10.69 ± 0.52	13.90 ± 0.23 ^e^	74.93 ± 0.13	10.00 ± 0.24 ^c^	21.29 ± 0.02 ^b^
	21	63.82 ± 0.91	9.33 ± 1.02	12.80 ± 0.27 ^f^	74.81 ± 0.16	9.69 ± 0.13 ^c^	21.34 ± 0.06 ^b^
	28	62.92 ± 0.15	10.54 ± 1.18	14.60 ± 0.17 ^d^	74.99 ± 0.25	8.56 ± 0.13 ^d^	21.41 ± 0.03 ^b^
14	14	64.78 ± 0.34	11.04 ± 0.73	10.87 ± 0.09 ^h^	74.62 ± 0.24	12.67 ± 0.19 ^b^	21.54 ± 0.03 ^b^
	21	63.88 ± 0.43	11.18 ± 0.24	12.57 ± 0.20 ^fg^	75.01 ± 0.21	13.31 ± 0.12 ^a^	21.38 ± 0.04 ^b^
	28	63.82 ± 1.17	11.17 ± 0.31	12.03 ± 0.18 ^g^	75.17 ± 0.06	13.33 ± 0.38 ^a^	20.76 ± 0.08 ^c^
Main effects							
Lipid level	6	63.83 ± 0.77	9.03 ± 0.13 ^y^	16.34 ± 0.38 ^x^	74.78 ± 0.08	7.14 ± 0.16 ^z^	21.37 ± 0.14
	10	63.60 ± 0.36	10.19 ± 0.52 ^x^	13.77 ± 0.29 ^y^	74.91 ± 0.10	9.41 ± 0.24 ^y^	21.35 ± 0.03
	14	64.16 ± 0.40	11.13 ± 0.24 ^x^	11.82 ± 0.26 ^z^	74.93 ± 0.12	13.11 ± 0.17 ^x^	21.23 ± 0.12
Starch level	14	63.93 ± 0.46	10.29 ± 0.39	14.16 ± 0.99	74.83 ± 0.10	10.04 ± 0.76 ^L^	21.25 ± 0.11
	21	63.84 ± 0.69	9.76 ± 0.48	13.82 ± 0.58	74.81 ± 0.10	10.12 ± 1.87 ^L^	21.37 ± 0.02
	28	63.83 ± 0.46	10.30 ± 0.46	13.96 ± 0.50	74.98 ± 0.11	9.50 ± 1.00 ^m^	21.33 ± 0.16
Two-way ANOVA (*p*-value)							
Lipid level		0.797	0.003	<0.001	0.511	<0.001	0.087
Starch level		0.991	0.499	0.153	0.459	0.002	0.239
Interaction		0.592	0.797	<0.001	0.235	0.001	<0.001

Values with different superscript letters in the each column were significantly different (*p* < 0.05). All data are expressed as means ± SE.

**Table 4:** Effects of varying dietary lipid and starch levels on serum parameters in hybrid grouper.

Lipid Level (%)	Starch Level (%)	TG (mmol/L)	TC (mmol/L)	TP (g/L)	ALT (U/L)	LDL-C (mmol/L)
6	14	1.56 ± 0.09 ^de^	1.51 ± 0.50	37.55 ± 0.21 ^a^	66.29 ± 1.95	0.19 ± 0.04
	21	1.60 ± 0.08 ^cde^	0.71 ± 0.08	35.86 ± 0.27 ^a^	68.64 ± 2.49	0.25 ± 0.07
	28	1.62 ± 0.16 ^cde^	0.96 ± 0.08	27.68 ± 1.26 ^cd^	70.80 ± 1.61	0.30 ± 0.01
10	14	2.04 ± 0.05 ^b^	1.25 ± 0.28	30.59 ± 1.27 ^bc^	74.88 ± 2.40	0.40 ± 0.08
	21	1.46 ± 0.03 ^e^	0.92 ± 0.07	24.80 ± 1.88 ^d^	75.60 ± 5.35	0.19 ± 0.01
	28	1.88 ± 0.08 ^bc^	1.10 ± 0.01	28.90 ± 2.41 ^bc^	66.89 ± 2.10	0.29 ± 0.04
14	14	1.83 ± 0.05 ^bcd^	1.29 ± 0.31	30.39 ± 0.54 ^bc^	72.30 ± 0.81	0.14 ± 0.02
	21	2.39 ± 0.16 ^a^	0.97 ± 0.10	32.01 ± 0.97 ^b^	69.27 ± 1.35	0.27 ± 0.02
	28	2.05 ± 0.02 ^b^	1.07 ± 0.08	31.42 ± 0.62 ^bc^	72.76 ± 1.59	0.34 ± 0.16
Main effects						
Lipid level	6	1.60 ± 0.06 ^z^	1.09 ± 0.19	33.70 ± 1.57 ^x^	68.58 ± 1.21	0.25 ± 0.03
	10	1.79 ± 0.09 ^y^	1.09 ± 0.10	28.09 ± 1.29 ^z^	72.46 ± 2.28	0.29 ± 0.04
	14	2.09 ± 0.10 ^x^	1.11 ± 0.11	31.27 ± 0.44 ^y^	71.44 ± 0.85	0.25 ± 0.06
Starch level	14	1.81 ± 0.08	1.35 ± 0.19 ^L^	32.84 ± 1.24 ^L^	71.16 ± 1.57	0.24 ± 0.05
	21	1.82 ± 0.15	0.87 ± 0.05 ^n^	30.89 ± 1.73 ^lm^	71.17 ± 2.07	0.24 ± 0.02
	28	1.85 ± 0.08	1.04 ± 0.04 ^lm^	29.33 ± 0.98 ^m^	70.15 ± 1.24	0.31 ± 0.05
Two-way ANOVA (*p*-value)						
Lipid level		<0.001	0.963	<0.001	0.173	0.656
Starch level		0.849	0.048	0.011	0.850	0.353
Interaction		<0.001	0.783	<0.001	0.081	0.113

TG, triglyceride; TC, total cholesterol; TP, total protein; ALT, alanine transaminase; LDL-C, low density lipoprotein cholesterol. Values with different superscript letters in the each column were significantly different (*p* < 0.05). Data are expressed as means ± SE.

**Table 5:** Effects of varying dietary lipid and starch levels on hepatic glycolipid metabolic enzyme activities in hybrid grouper.

Lipid Level (%)	Starch Level (%)	FAS (pmol/g)	FBP (ng/g)	PEPCK (U/g)	PK (U/gprot)	PFK (U/mg)
6	14	53.37 ± 0.78	0.22 ± 0.01 ^cd^	0.93 ± 0.16	28.05 ± 2.07 ^b^	318.33 ± 39.95
	21	49.14 ± 1.25	0.20 ± 0.01 ^d^	0.80 ± 0.12	49.21 ± 3.18 ^a^	357.33 ± 39.50
	28	46.98 ± 3.74	0.21 ± 0.01 ^d^	0.75 ± 0.08	51.99 ± 5.58 ^a^	355.50 ± 30.05
10	14	44.19 ± 4.05	0.22 ± 0.01 ^bcd^	0.98 ± 0.05	28.35 ± 3.81 ^b^	290.35 ± 13.94
	21	49.14 ± 0.88	0.26 ± 0.02 ^abc^	0.70 ± 0.02	46.21 ± 6.90 ^a^	315.67 ± 10.93
	28	51.84 ± 2.24	0.29 ± 0.02 ^a^	0.81 ± 0.02	38.34 ± 7.64 ^ab^	356.33 ± 20.35
14	14	45.72 ± 3.74	0.23 ± 0.01 ^bcd^	0.90 ± 0.08	29.01 ± 8.59 ^b^	226.10 ± 19.84
	21	49.41 ± 2.28	0.26 ± 0.02 ^ab^	0.78 ± 0.06	21.01 ± 2.53 ^b^	276.50 ± 22.49
	28	42.84 ± 0.68	0.26 ± 0.01 ^abc^	0.61 ± 0.02	22.88 ± 2.76 ^b^	206.00 ± 9.40
Main effects						
Lipid level	6	49.86 ± 1.49	0.21 ± 0.01 ^y^	0.83 ± 0.07	43.09 ± 4.25 ^x^	343.72 ± 19.51 ^y^
	10	48.42 ± 1.76	0.26 ± 0.01 ^x^	0.83 ± 0.04	37.63 ± 4.09 ^x^	320.78 ± 12.36 ^x^
	14	45.99 ± 1.67	0.25 ± 0.01 ^x^	0.76 ± 0.05	24.86 ± 2.96 ^y^	236.20 ± 13.86 ^x^
Starch level	14	47.79 ± 2.15	0.22 ± 0.01 ^m^	0.94 ± 0.05 ^L^	28.47 ± 2.78 ^m^	278.26 ± 19.18
	21	49.23 ± 0.94	0.24 ± 0.01 ^lm^	0.76 ± 0.04 ^m^	38.80 ± 5.03 ^L^	316.50 ± 17.83
	28	47.25 ± 1.82	0.25 ± 0.01 ^L^	0.72 ± 0.04 ^m^	38.29 ± 5.08 ^L^	305.94 ± 27.28
Two-way ANOVA (*p*-value)						
Lipid level		0.217	<0.001	0.517	0.002	<0.001
Starch level		0.627	0.034	0.009	0.046	0.191
Interaction		0.083	0.047	0.508	0.046	0.290

FAS, fatty acid synthase; FBP, fructose-1,6-bisphosphatase; PEPCK, phosphoenolpyruvate carboxykinase; PK, pyruvate kinase; PFK, phosphofructokinase. Values with different superscript letters in the each column were significantly different (*p* < 0.05). Data are expressed as means ± SE.

## Data Availability

The data that support the findings of this study are available from the corresponding author upon reasonable request.
